# Rapid and reliable diagnosis of murine myeloid leukemia (ML) by FISH of peripheral blood smear using probe of PU. 1, a candidate ML tumor suppressor

**DOI:** 10.1186/1755-8166-1-22

**Published:** 2008-10-16

**Authors:** Reiko Kanda, Satsuki Tsuji, Yasushi Ohmachi, Yuka Ishida, Nobuhiko Ban, Yoshiya Shimada

**Affiliations:** 1 Research Center for Radiation Protection, National Institute of Radiological Sciences, Chiba, Japan; 2 Fundamental Technology Center, National Institute of Radiological Sciences, Chiba, Japan; 3 Laboratory of Environmental Health Science, Oita University of Nursing and Health Sciences, Oita, Japan

## Abstract

**Background:**

Murine myeloid leukemia (ML) provides a good animal model to study the mechanisms of radiation-induced leukemia in humans. This disease has been cytogenetically characterized by a partial deletion of chromosome 2 with G-banding. For the rapid diagnosis of ML, this study reports a FISH method using spleen cells and peripheral blood smears from ML mice exposed to gamma rays and neutrons with PU.1, a candidate ML tumor suppressor, as a probe.

**Results:**

Among mice that were tentatively diagnosed with ML by clinical findings and blood smear examination, 85% carried spleen cells showing the loss of PU.1 although the frequency of these abnormal cells varied among individuals. Mice with very low frequencies of cells showing the loss of one copy of PU.1 (one-PU.1 frequency) were later diagnosed pathologically not with ML but with blastic or eosinophilic leukemia. Some neutron-irradiated mice had cells showing translocated PU.1, although no pathological features differentiated these ML mice from ML mice expressing the simple loss of PU.1.

The one-PU.1 frequency can be detected from spleen metaphase cells, spleen interphase cells, and blood smears. There was a good correlation between the one-PU.1 frequency in spleen metaphase cells and that in spleen interphase cells (r = 0.96) and between one-PU.1 frequency in spleen interphase cells and that in blood cells (r = 0.83).

**Conclusion:**

The FISH method was capable of detecting aberration of copy number of the PU.1 gene on murine chromosome 2, and using a peripheral blood smear is more practical and less invasive than conventional pathological diagnosis or the cytogenetic examination of spleen cells.

## Background

Murine myeloid leukemia (ML) provides a good animal model to study the mechanisms of radiation-induced leukemia in humans because it is thought to be similar to the cancer type that has shown increased incidence among atomic-bomb survivors [1]. This disease is cytogenetically characterized by a partial deletion of chromosome 2 [2-4]. Silver et al. [5] mapped the minimal deleted region to a 1.0 cM region homologous to human chromosome segment 11p11-12. Recently, the critical hematopoietic-specific transcription factor PU.1 (also known as SPI-1), which is located within this region, is proposed as a ML tumor suppressor [5-7].

It is very important for the diagnosis of murine ML to check for aberrations on chromosome 2, as in the case of Philadelphia chromosome for the diagnosis of human ML. For this purpose, murine chromosomes are analyzed using G-banding, Q-banding, and FISH techniques using preparations of bone marrow or spleen cells. However, in mice the time course of chromosomal changes cannot be surveyed for individuals during radiation leukemogenesis because mice must be killed for tissue sampling.

The present study attempted to detect the aberration on murine chromosome 2 with a probe against the PU.1 gene using peripheral blood smears, and compared these aberration frequencies with those obtained from metaphase and interphase cells isolated from spleens. As a result, the frequency of cells showing the loss of one copy of PU.1 obtained by this method was very close to that from slides prepared traditionally using spleen cells, and this new method was useful for the rapid diagnosis of the detailed classification of ML in living mice.

The validation of this method to diagnose murine ML is discussed as well as its prospective application for the diagnosis of human ML.

## Results and discussion

### Frequency of PU.1 abnormalities in radiation-induced ML mice

**Figure 1:**
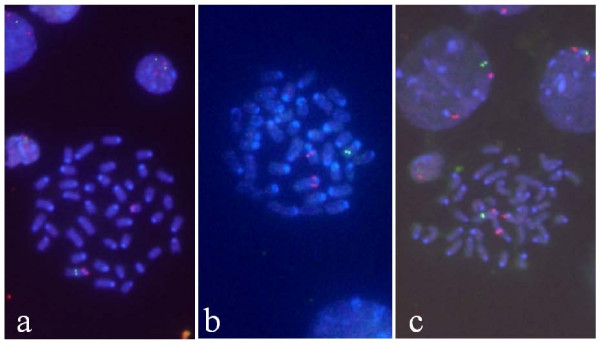
**Spleen cells of radiation-induced myeloid leukemia mice observed by FISH using probes against PU.1 (green) and the centromere of chromosome 2 (red)**. G1R2 cells with only one signal of PU.1 on chromosome 2 (a) and on another chromosome (b). Cells with 3 centromere signals suggested numerical abnormality (c).

Interphase FISH analyses using PU.1 were performed using spleen cells of ML-like mice that were sorted by clinical findings and blood smear examination. Cells showing the loss of one copy of the PU.1 gene due to the partial deletion of chromosome 2 had 1 PU.1 signal (green) and 2 centromere signals (red), and were named G1R2 cells (Fig. 1a,b). Among 11 gamma-irradiated ML-like mice and 9 neutron-irradiated mice, 85% carried G1R2 cells, which was consistent with the previous finding that more than 90% of acute ML mice carry aberrations of chromosome 2 [2,4,8]. In detail, 9 mice carried G1R2 cells among 11 gamma-irradiated ML-like mice, and 8 mice carried G1R2 cells among 9 neutron-irradiated mice. Regarding 17 mice that carried G1R2 cells, the percentages of G1R2 cells among all cells examined varied from 54 to 100%.

### Comparison of metaphase and interphase analyses of spleen cells

The results of FISH analysis were compared between metaphase and interphase cells for 7 gamma-irradiated ML-like mice and 6 neutron-irradiated mice whose mitotic indices were relatively high (Table 1). In 8 mice, all the examined cells were G1R2 cells. One mouse (ID: c090) exhibited that both copies of chromosome 2 were apparently normal in all cells examined. In 4 other mice, mosaics of normal and aberrant cells were observed.

**Table 1:** Cell distribution with aberration of PU.1 in the spleens of radiation-induced myeloid leukemia mice

		No of metaphase cells with					No of interphase cells with		
		
		2 PU.1 signals			1 PU.1 signal (1 deleted)				
					
Mouse ID	Exposure	Both normal	1 trans -located	2 trans -located	1 normal	1 trans -located	2 PU.1 signals	1 PU.1 signal (1 deleted)	
b007	Gamma	1	0	0	19	0	1	19	Mosaic
b021	Gamma	0	0	0	14	0	0	50	All G1R2
b154	Gamma	0	0	0	18	0	2	48	Mosaic
c090	Gamma	15	0	0	0	0	50	0	All normal
d040	Gamma	0	0	0	9	0	0	50	All G1R2
e036	Gamma	0	0	0	20	0	0	50	All G1R2
e127	Gamma	0	0	0	20	0	0	50	All G1R2

h060	Neutron	0	0	0	7	0	0	50	All G1R2
h061	Neutron	0	12	0	8	0	23	27	Mosaic
h070	Neutron	0	0	0	7	0	0	50	All G1R2
h121	Neutron	0	0	2	0	18	9	41	Mosaic
h124	Neutron	0	0	0	20	0	0	50	All G1R2
j018	Neutron	0	0	0	8	0	0	20+30*	All G1R2

*Cells with 1 signal of PU.1 and 3 centromere signals

Metaphase analysis of one mouse (ID: h061) showed that one PU.1 signal was translocated in 60% of cells examined. Metaphase analysis of another mouse (ID: h121) showed that both PU.1 signals were translocated in 10% of cells, and one was translocated and the other was lost in 90% of cells (Fig. 1b). In a further mouse (ID: j018), 60% of cells had 1 signal of PU.1 and 3 signals from the centromere (Fig. 1c), suggesting a numerical abnormality. These complex abnormalities were observed in neutron-irradiated ML-like mice; however, no pathological features of differentiated ML mice expressing complex abnormalities were identified from those expressing the simple loss of one copy of PU.1.

As shown in Table 1, interphase FISH analysis data were qualitatively consistent with metaphase data, and the frequency of complex abnormalities might be higher in neutron-irradiated mice than gamma-irradiated mice. In general, metaphase FISH analysis provides more information regarding complex chromosome aberrations than interphase analysis so the latter cannot entirely replace the former. In many cases, the mitotic indices of spleen cells and bone marrow cells were considerably lower (less than 1%), which made metaphase analysis difficult. The complex abnormalities of PU.1, which could be observed only by metaphase analysis, did not show the relevance to pathological features. To substitute for pathological diagnosis of ML, therefore, assessment for presence of G1R2 cells might be more important than the observation of complexity of aberrations.

### Frequency of cells with 1 PU.1 signal and 2 centromere signals

**Figure 2:**
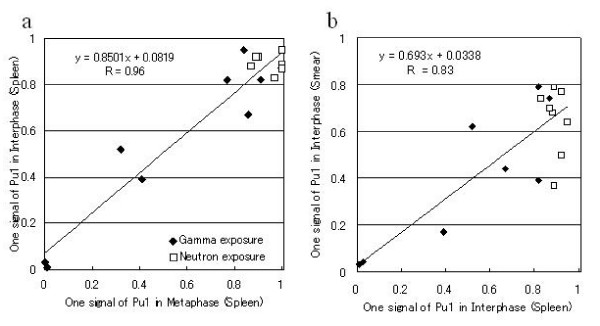
**Comparison of the frequency of cells with one signal of PU.1 and 2 signals of the centromere of chromosome 2 in radiation-induce myeloid leukemia mice**. Correlation between the frequency of cells showing a loss of one copy of PU.1 in spleen metaphase cells and that in spleen interphase cells (a) and between the frequencies in spleen interphase cells and that in blood cells (b). The some points were superimposed.

Next, metaphase and interphase analyses were compared quantitatively. In this experiment, mice were diagnosed with ML not only by clinical findings and blood smear examination but also by flow cytometry and pathological investigations to clarify the relation of frequency of G1R2 cells and clinical diagnosis. The percentages of G1R2 cells varied from 0 to 100% (Fig. 2), but cells with 2 red and 0 green signals, i.e. with the loss of both PU.1 signals, were not observed. There was a good correlation between the percentage of cells with one PU.1 signal in the metaphase and interphase specimens (r = 0.96), which suggested the high reliability of the results obtained by interphase FISH.

Among 17 mice, two showed very low frequencies of G1R2 cells and very high WBC counts (about 2000 × 10^2^/μl). Later, the pathological examination indicated the possible diagnosis of blastic-type and eosinophilic leukemia. The frequency of G1R2 cells in spleen cells may be a useful marker to identify false-positive ML.

### FISH data of peripheral blood smears

**Figure 3:**
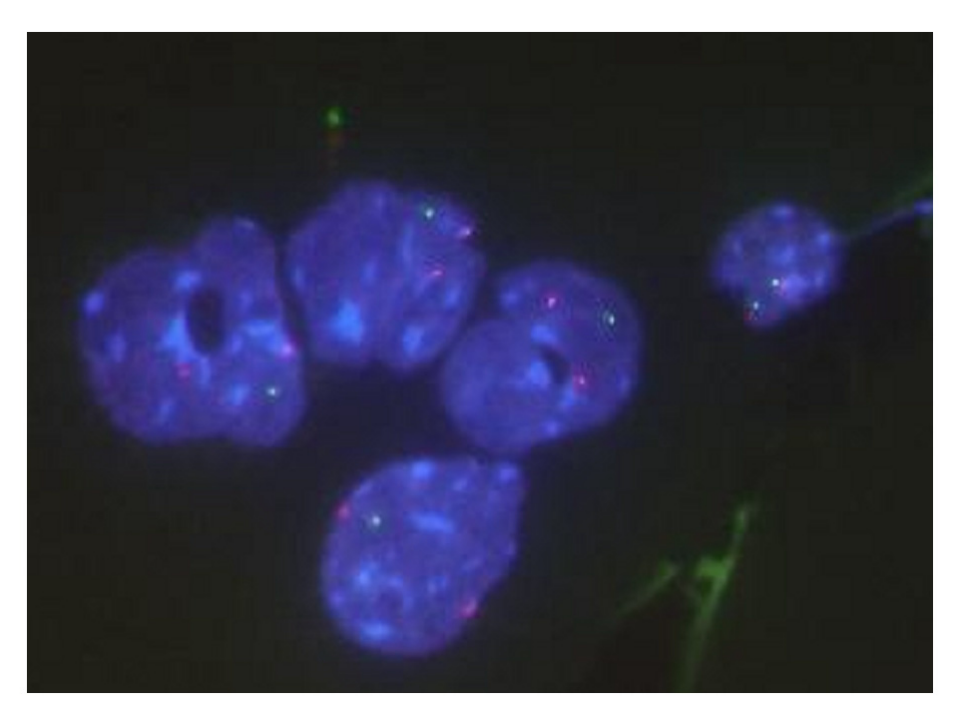
Blood smear specimen of radiation-induced myeloid leukemia mice observed by FISH using probes against PU.1 (green) and the centromere of chromosome 2 (red).

Interphase specimens from peripheral blood smears were thicker than those from spleen cells due to the different preparation methods so FISH signals were detected by Z-stack function or by manually varying the focus (Fig. 3). It was attempted to distinguish granulocytic cells from erythroblasts, but this was found to be impossible. There was a good correlation between the percentage of cells with one signal of PU.1 in blood smears and that for interphase spleen specimens (r = 0.83).

FISH analysis of a blood smear using PU.1 has good potential as a method for the rapid diagnosis of ML that was comparable to pathological diagnosis, but the latter is time-consuming and labor intensive. Cells with the loss of one copy of PU.1 can be counted the day after blood sampling; blood smear preparation, FISH hybridization, washing, and microscopic observation require 1 hour, overnight, 3 hours, and a few hours, respectively. In addition, FISH analysis does not require cytogenetic skill or highly-specialized equipment.

This technique can be useful for understanding radiation leukemogenesis. The partial deletion of chromosome 2 by radiation is suspected to be an initiating event of radiation leukemogenesis [9]. FISH analysis using blood smears can use living mice so that the time course of chromosomal changes can be surveyed for individuals during leukemogenesis. In addition, in humans, the sampling of bone marrow cells is painful for frail patients. Trials have been started to clarify the clinical correlation of chromosome changes in blood cells in chronic leukemia [10,11]. For these cases, less invasive sampling for cytogenetic analysis has advantages.

## Conclusion

The FISH method for detecting aberrations of the PU.1 gene on murine chromosome 2 using peripheral blood smears may be used as a rapid diagnostic method for the detailed classification of ML. This method is more practical and less invasive than conventional pathological diagnosis and for cytogenetic examination of spleen cells.

## Methods

### Animals

Male C3H mice were bred in the animal facility of the National Institute of Radiological Sciences, Chiba, Japan. They were housed in an air-conditioned room (23 ± 1°C) with a relative humidity of 50 ± 5% under an alternating 12-h light/dark schedule (lights on at 7:00 a.m.). Mice were fed a standard commercial laboratory diet and given chlorinated water *ad libitum*. Mice received a dose of 3 Gy of ^137^Cs γ-rays or neutrons generated from the NIRS cyclotron, using the deuteron-beryllium reaction. The animals were observed for their whole life span. When symptoms of leukemia, such as a palpable spleen, wan palms, respiratory distress, etc., became clear in the terminal stage of the disease, the mice were anesthetized with diethyl ether and tissues were sampled. ML-like mice were sorted by histological examination of peripheral blood smears, blood cell counts, and/or flow cytometric examination, and the comprehensive diagnosis in each case was finally established from histopathological investigations of tissue/organs [12]. All animal experiments were carried out with permission of and under regulation of the Institutional Committee for Animal Safety and Welfare at our institute. The smear preparations of peripheral blood were made conventionally; one drop of blood was placed on a clean slide, spread with another slide and dried.

### Isolation of lymphocytes

The spleen cells were suspended mechanically in a dish containing RPMI 1640 medium and filtered through a 100 μm nylon filter (BD Falcon, MA, USA). Except for spleen cells for flow cytometry, cells were centrifuged, resuspended in a 0.075 M KCl hypotonic solution with 0.025 μg/ml colcemid, kept at 37°C for 35 min, and then fixed with methanol-acetic acid (3:1). Air-dried slides were made in warm and humid conditions. The mitotic indices of spleen cells were 0.5–1.5%.

The surface markers of spleen cells of some mice showing symptoms of leukemia were analyzed by flow cytometry using mouse monoclonal antibodies, and individuals with positive cells for Mac-1 and Gr-1 in the spleen were sorted as "ML mouse candidates".

### Microscopic observation

FISH analysis of peripheral blood smears and spleen cells was performed to identify deletions on chromosome 2. When chromosome preparations of spleen cells were compared with those of bone marrow cells; mitotic indices of the former and the latter were 1.15 ± 0.16% and 0.71 ± 0.11%, respectively (n = 14), and the chromosomes of spleen cells were easier to spread than those of bone marrow cells. Hayata [13] reported that the percentage of leukemic cells among normal cells, the abnormal chromosome number and the number of clones were almost the same between spleens and bone marrow; therefore, the present study analyzed chromosomes using spleen samples not bone marrow samples. FISH probes covering PU.1 and the centromere of chromosome 2 were labeled directly with Spectrum Green and Spectrum Orange (Vysis, Downers Grove, IL, USA), respectively [14]. For FISH analysis, fresh peripheral blood smears were fixed with methanol-acetic acid (3:1), dried, stored at 4°C, and then treated with RNase just before hybridization [15]. The slides were hybridized with probes following the manufacturer's instructions. The slides were counterstained with DAPI in Vectorshield mounting medium (Vector Laboratories, Burlingame, CA, USA). FISH analysis was performed for more than 30 cells in each case by using an all-in-one type fluorescence microscope BZ-8000 (Keyence Japan, Osaka, Japan). Cells with 2 red signals, i.e. having 2 centromeres of chromosome 2, were mainly counted. For each case, more than 7 and 30 cells were scored for metaphase and interphase analysis, respectively, according to the protocol of Yanagi et al. [10].

## Competing interests

The authors declare that they have no competing interests.

## Authors' contributions

RK and ST carried out the cytogenetic studies and drafted the manuscript. YO carried out animal experiments and pathological analysis. YI carried out the flow cytometric analysis. NB developed the FISH technique using the PU.1 probe. YS participated in the design of the study. All authors read and approved the final manuscript.

## References

[B1] Preston DL, Kusumi S, Tomonaga M, Izumi S, Ron E, Kuramoto A, Kamada N, Dohy H, Matsuo T, Nonaka H, Thompson DE, Soda M, Mabuchi K (1994). Cancer incidence in atomic bomb survivors. Part III. Leukemia, lymphoma and multiple myeloma, 1950–1987. Radiat Res.

[B2] Hayata I, Seki M, Yoshida K, Hirashima K, Sado T, Yamagiwa J, Ishihara T (1983). Chromosomal aberrations observed in 52 mouse myeloid leukemias. Cancer Res.

[B3] S Breckton G, Silver A, Cox R (1991). Radiation-induced chromosome 2 breakage and the initiation of murine radiation acute myeloid leukaemogenesis. J Radiat Res.

[B4] Rithidech K, Dunn JJ, Bond VP, Gordon CR, Cronkite EP (1999). Characterization of genetic instability in radiation- and benzene-induced murine acute leukemia. Mutat Res.

[B5] Silver A, Moody J, Dunford R, Clark D, Ganz S, Bulman R, Bouffler S, Finnon P, Meijne E, Cox R (1999). Molecular mapping of chromosome 2 deletions in murine radiation-induced AML localizes a putative tumor suppressor gene to a 1.0 cM region homologous to human chromosome segment 11p11-12. Genes Chromosomes Cancer.

[B6] Cook WD, McCaw BJ, Herring C, John DL, Foote SJ, Nutt SL, Adams JM (2004). PU.1 is a suppressor of myeloid leukemia, inactivated in mice by gene deletion and mutation of its DNA binding domain. Blood.

[B7] Walter MJ, Park JS, Ries RE, Lau SK, McLellan M, Jaeger S, Wilson RK, Mardis ER, Ley TJ (2005). Reduced PU.1 expression causes myeloid progenitor expansion and increased leukemia penetrance in mice expressing PML-RARalpha. Proc Natl Acad Sci USA.

[B8] Trakhtenbrot L, Krauthgamer R, Resnitzky P, Haran-Ghera N (1988). Deletion of chromosome 2 is an early event in the development of radiation-induced myeloid leukemia in SJL/J mice. Leukemia.

[B9] Jawad M, Cole C, Zanker A, Lo P, Fitch S, Plumb M (2006). Evidence for clustered tumour suppressor gene loci on mouse chromosomes 2 and 4 in radiation-induced acute myeloid leukaemia. Int J Radiat Biol.

[B10] Yanagi M, Shinjo K, Takeshita A, Tobita T, Yano K, Kobayashi M, Terasaki H, Naoe T, Ohnishi K, Ohno R (1999). Simple and reliably sensitive diagnosis and monitoring of Philadelphia chromosome-positive cells in chronic myeloid leukemia by interphase fluorescence in situ hybridization of peripheral blood cells. Leukemia.

[B11] Sindelárová L, Michalová K, Zemanová Z, Ransdorfová S, Brezinová J, Peková S, Schwarz J, Karban J, Cmunt E (2005). Incidence of chromosomal anomalies detected with FISH and their clinical correlations in B-chronic lymphocytic leukemia. Cancer Genet Cytogenet.

[B12] Ohmachi Y, Hiraoka T, Ishida Y, Ogiu T (2004). Neutron biological effects research at NIRS. Proc of the Japan-France Workshop on Radiobiology and Isotopic Imaging.

[B13] Hayata I, Ishihara T, Sasaki MS, Alan R (1983). Partial deletion of chromosome 2 in radiation-induced myeloid leukemia in mice. Radiation-induced Chromosome Damage in Man.

[B14] Ban N, Yoshida K, Aizawa S, Wada S, Kai M (2002). Cytogenetic Analysis of radiation-induced leukemia in trp53-deificient C3H/He mice. Radiat Res.

[B15] Hayata I, Tabuchi H, Furukawa A, Okabe N, Yamamoto M, Sato K (1992). Robot system for preparing lymphocyte chromosome. J Radiat Res.

